# Flexible trial design in practice – dropping and adding arms in STAMPEDE: a multi-arm multi-stage randomised controlled trial

**DOI:** 10.1186/1745-6215-12-S1-A3

**Published:** 2011-12-13

**Authors:** Matthew R Sydes, Nicholas D James, Malcolm D Mason, Noel W Clarke, Claire Amos, John Anderson, Johann de Bono, David P Dearnaley, John Dwyer, Gordana Jovic, Alastair Ritchie, Martin Russell, Karen Sanders, George Thalmann, Mahesh KB Parmar

**Affiliations:** 1 MRC Clinical Trials Unit, London, UK; 2 CRUK Institute for Cancer Studies, University of Birmingham, Birmingham, UK; 3 School of Medicine, Cardiff University, Cardiff, UK; 4 The Christie and Salford Royal Hospitals Foundations Trusts, Manchester, UK; 5 The Royal Hallamshire Hospital, Sheffield, UK; 6 The Institute for Cancer Research and Royal Marsden Hospital, Sutton, UK; 7 Prostate Cancer Support Federation, Stockport, UK; 8 Beatson West of Scotland Cancer Centre, Glasgow, UK; 9 Inselspital, Bern, Switzerland

## Objectives

STAMPEDE is a multi-centre, randomised controlled trial designed with novel multi-arm, multi-stage (MAMS) methods. Here we: **(1)** describe the methodological and practical issues arising with the stopping of recruitment to some trial arms following an intermediate analysis and **(2)** describe the issues surrounding the addition of new research arms during the trial.

## Methods

The trial recruits men with locally advanced or metastatic prostate cancer starting standard long-term hormone therapy. There are 5 research arms and 1 control arm. The trial has a pilot stage assessing safety and feasibility, 3 intermediate “activity” stages (I-III) where the outcome measure is failure-free survival (FFS) and one final “efficacy” stage (IV) with overall survival as primary outcome measure. At the end of each stage, each research arm is formally compared pairwise to the control arm. Accrual of further patients is discontinued early for any research arm either not showing sufficient evidence of activity or with adverse safety considerations; accrual continues to arms showing activity with acceptable safety. The stopping guideline compares the treatment effect against a pre-defined cut-off value using the hazard ratio when the hazards are proportional and restricted-mean survival time otherwise. This interim hurdle becomes increasingly stringent stage-by-stage. The addition of new research arm(s) can be actively considered when sufficiently interesting agents emerge. New research arms are compared only to contemporaneously-recruited control arm patients using the same intermediate guidelines in a time-delayed manner. The addition of new research arms is independent of any of the original research arms stopping accrual early subject to adequate recruitment to support the overall trial aims.

## Results

**(1)** After the second intermediate activity analysis (March-2011), the IDMC recommended and the Trial Steering Committee ratified discontinuation of recruitment to two research arms for lack-of-sufficient activity. Nearly 100 recruiting centres in UK and Switzerland had to promptly implement the changes. Detailed advanced preparation meant that activation was swift and recruitment continued seamlessly into Activity Stage III; MHRA and REC approval was not required because this was already included in the trial design. **(2)** An application to add a new research arm has been successfully approved by the CRUK’s CTAAC funding committee and accepted by the relevant industry partner. Details on the methodological and practical issues and implementation of these changes will be presented.

## Conclusions

The STAMPEDE experiences shows that recruitment to MAMS trial is achievable and that mid-flow changes to trial design are practicable with good planning.

